# Editorial: Inflammation in ischemic stroke and novel therapeutic strategies for stroke treatment

**DOI:** 10.3389/fneur.2022.1071557

**Published:** 2022-11-18

**Authors:** Hany E. Marei, Changjun Yang, Carlo Cenciarelli, Assia Jaillard

**Affiliations:** ^1^Department of Cytology and Histology, Faculty of Veterinary Medicine, Mansoura University, Mansoura, Egypt; ^2^Department of Neuroscience, University of Florida, Gainesville, FL, United States; ^3^Institute of Translational Pharmacology (IFT)-CNR, Rome, Italy; ^4^Université Grenoble Alpes Saint Martin d'Hères, Grenoble, France

**Keywords:** ischemic stroke, neuroinflammation, blood–brain barrier, oxidative stress, stem cells

Stroke is one of the leading causes of death and long-term disability worldwide (1). An ischemic stroke occurs when a major cerebral blood vessel becomes blocked, depriving downstream tissue of oxygen and nutrients and resulting in cell death within minutes at the core of the infarct. Dying cells release pro-inflammatory signals, which activate resident astrocytes/microglia and initiate immune cell infiltration from the periphery into the damaged tissue, contributing to blood–brain barrier disruption and exacerbating cell death in a process known as secondary inflammation, which can last for days to weeks after the initial insult (2). The premise of tissue repair in acute ischemic stroke is the proper termination of cell-death-induced neural inflammation (AIS). Macrophages scavenge cell corpses and produce inflammatory mediators that coordinate immune responses (3).

Stem cell therapy is a hot research area and a promising clinical therapeutic modality for ischemic stroke. Cell-engineering approaches are expected to usher in a new generation of stem cell-based therapies, greatly expanding their therapeutic utility for a variety of traumatic and neurodegenerative diseases, including ischemic stroke (4).

This Research Topic includes five manuscripts that highlight current knowledge and future directions in the role of neuroinflammation and the potential use of cell-based therapies in ischemic stroke. Zhou et al. look into whether L-4F displays neurorestorative benefits in the ischemic brain and the underlying molecular mechanisms after stroke in type 2 diabetes mellitus (T2DM). They concluded that administering L-4F post-stroke may provide a restorative strategy for type 2 diabetes mellites (T2DM)-stroke by promoting neurovascular and white matter (WM) remodeling. Reducing neuroinflammation in the injured brain may aid the restorative effects of L-4F that are not mediated by the ABCA1 signaling pathway (Zhou et al.). Yu et al. have looked at the effects of metformin, rapamycin, and nicotinamide mono nucleotide (NMN) on cognitive function, white matter integrity, microglial response, and phagocytosis in a rat model of vascular cognitive impairment (VCI) caused by bilateral common carotid artery occlusion (BCCAO). According to the findings, metformin, rapamycin, or NMN may protect or mitigate cognitive impairment and WMLs by modifying microglial polarization and inhibiting phagocytosis. The findings could pave the way for a new approach to VCI treatment (Yu et al.). Zhao et al. compared the remote ischemic postconditioning (RIPostC) group to a control group in a meta-analysis of eligible randomized controlled trials in patients with ischemic stroke. They concluded that RIPostC is safe and effective, with a positive cerebral protective effect in patients with ischemic stroke, and that large-sample, multicenter trials are needed to validate RIPostC's cerebral protective effect in the future (5). Custodia et al. review the most recent advances in preclinical and clinical research on the use of endothelial progenitor cells (EPCs) after stroke, not only as a single treatment but also in combination with novel therapeutic approaches. Following cerebrovascular damage, EPCs can repair damaged vessels as well as generate new ones. EPCs are circulating cells that have endothelial cell and adult stem cell characteristics, including the ability to differentiate into mature endothelial cells and self-renew. Furthermore, EPCs have the advantage of already being present in healthy conditions as circulating cells that participate in endothelial maintenance in a direct and paracrine manner. Based on clinical data demonstrating a better neurological and functional outcome in ischemic stroke patients with higher levels of circulating EPCs, novel and promising therapeutic approaches would be EPCs-promoting pharmacological treatments as well as EPCs-based therapies (5). In rodent stroke models, Satani et al. have proposed that systemic administration of marrow stromal cells (MSCs) causes the release of a wide range of factors that mediate recovery. In this study they have investigated the immunomodulatory interactions between MSCs and peripheral blood-derived monocytes (Mo) obtained from acute stroke patients. This study found MSCs had a differential effect on Mo derived from acute stroke patients vs. those derived from healthy controls, suggesting that immunomodulation of immune cells may represent a therapeutic target for MSCs in patients with acute stroke (Satani et al.).

## Author contributions

All authors listed have made a substantial, direct, and intellectual contribution to the work and approved it for publication.

## Conflict of interest

The authors declare that the research was conducted in the absence of any commercial or financial relationships that could be construed as a potential conflict of interest.

## Publisher's note

All claims expressed in this article are solely those of the authors and do not necessarily represent those of their affiliated organizations, or those of the publisher, the editors and the reviewers. Any product that may be evaluated in this article, or claim that may be made by its manufacturer, is not guaranteed or endorsed by the publisher.
